# Waiting time variation in Early Intervention Psychosis services: longitudinal evidence from the SEPEA naturalistic cohort study

**DOI:** 10.1007/s00127-017-1343-7

**Published:** 2017-02-18

**Authors:** James B. Kirkbride, Y. Hameed, L. Wright, K. Russell, C. Knight, J. Perez, P. B. Jones

**Affiliations:** 1 PsyLife Group, Division of Psychiatry, UCL, 6th Floor Maple House, 149 Tottenham Court Road, London, W1T 7NF UK; 2 Department of Psychiatry, University of Cambridge, Cambridge, CB2 0SZ UK; 3 Norfolk and Suffolk Foundation Trust, Norwich, Norfolk NR6 5BE UK; 4 Cambridgeshire and Peterborough Foundation Trust, NIHR Collaboration for Leadership in Applied Health Research and Care (CLAHRC) East of England, Cambridge, Cambridgeshire CB21 5EF UK

**Keywords:** Mental health services, Psychotic disorders, Early intervention (education), Health services research, Cohort studies

## Abstract

**Purpose:**

Early Intervention Psychosis [EIP] services have gained traction internationally, but are currently undergoing various forms of reconfiguration. In England, such services are now mandated to ensure 50% of accepted referrals commence care within 14 days, but no empirical evidence exists. We sought to estimate waiting times to EIP services in a large, representative epidemiological cohort in England, and investigate possible reasons for any variation.

**Methods:**

We estimated median waiting time from referral to acceptance by EIP services and investigated whether this varied by clinical, demographic or neighbourhood-level factors, amongst 798 participants, 16–35 years old, presenting to six EIP services over 3.5 years in a defined catchment area serving 2.5 million people. We used parametric survival analysis to inspect variation in waiting times (in days).

**Results:**

Median waiting time was 15 days (interquartile range 7–30), although this varied across services (*p* < 0.01). Waiting times increased over the case ascertainment period by an average of 4.3 days (95% CI 1.3, 6.2; *p* < 0.01). Longer waiting times were associated with greater diagnostic uncertainty, indexed by an organic presentation (+ 9.1 days; 95% CI 1.9, 16.6; *p* < 0.01), polysubstance abuse (+ 2.6; 0.6, 3.9; *p* < 0.01), absence of psychotic disorder (+1.8; −0.1, 3.0; *p* = 0.05) and insidious onset (+1.8; −0.1, 3.0; *p* = 0.06). Waiting times did not vary by most demographic or neighbourhood-level characteristics.

**Conclusions:**

EIP services operate close to new waiting time standards in England, with little systematic variation by sociodemographic position. However, waiting times increased over the study period, coinciding with substantial service reorganisation. Longer waiting times associated with greater diagnostic uncertainty highlight opportunities to reduce delays in certain clinical groups at initial referral.

**Electronic supplementary material:**

The online version of this article (doi:10.1007/s00127-017-1343-7) contains supplementary material, which is available to authorized users.

## Introduction

Early Intervention in Psychosis [EIP] services offer a multi-disciplinary package of care for people experiencing their first episode of psychosis, underpinned by evidence that reducing the duration of untreated psychosis [DUP] leads to better clinical, functional and social outcomes for people experiencing psychosis [1]. Recent interest in EIP service provision has focussed on how best to deliver effective, timely and appropriate care for people experiencing psychiatric distress. For example, in Australia, services have moved towards a youth mental health model [2], emphasising the need for clinical staging during critical periods of adolescence to prevent a range of psychiatric morbidities. In Denmark [3, 4], Norway [5, 6], Hong Kong [7] and Canada [8] emphasis has focussed on identifying the optimum duration of intervention required to sustain medium- and long-term beneficial outcomes, while new services are currently gaining traction in the United States [9]. In England, the recognition that parity of esteem between mental and physical health conditions [10, 11] should be core to healthcare provision has led the Department of Health and NHS England to make policy commitments to improve access and waiting times to a variety of mental health services, including early intervention in psychosis. In April 2016, new “Access and Waiting Time Standard” came into force [12], mandating that at least half of all referrals to EIP services should commence a NICE-concordant package of care for psychosis within two weeks of referral [13], with a commitment to raise this standard to 60% by 2020/21. Efforts to reduce EIP waiting times are also concomitant with shortening the duration of untreated psychosis [DUP] in people in their first episode of psychosis [FEP], since delays within the mental health system contribute substantially to overall DUP [14, 15]. Despite this, no empirical evidence exists about the current magnitude of waiting times in EIP services, or whether these vary by clinical, demographic, environmental or service-level factors.

### Aims of the study

We sought to address this fundamental knowledge gap using data from a large, epidemiologically complete cohort of participants presenting to EIP services in the East of England, as part of the Social Epidemiology of Psychoses in East Anglia [SEPEA] study [16]. Given the lack of empirical evidence on this topic we held no a priori expectation about the magnitude of median waiting times in an EIP context. However, we hypothesised that waiting times would vary according to clinical presentation, with people presenting with more complex clinical psychopathologies at first referral having longer waiting times (indexed by an insidious (vs. acute) mode of onset, longer duration of untreated illness or more affective or non-psychotic phenotypes at initial presentation).

## Materials and methods

### Design and setting

The SEPEA study is a naturalistic cohort of all people, aged 16–35 years, who were referred, accepted and met epidemiological and clinical criteria for FEP in six EIP services over a 3.5-year ascertainment period in a defined catchment area in East Anglia, serving a total population of about 2.4 million people (4.5% of the 2011 English population). The study originally investigated variation in the incidence of clinically relevant psychotic disorders [16]. Here, we included 798 participants referred and accepted by EIP services from 1,005 initial referrals with suspected psychotic symptoms. This incepted sample included 687 (86.1%) incidence participants, who met clinical and epidemiological criteria for the study, as well as 111 (13.9%) people accepted by EIP services, but who were later found not to have sufficient symptomatology for psychotic disorder (*N* = 94; 11.8%) or who had an organic basis to their disorder (*N* = 17; 2.1%). The remaining 207/1005 participants who were referred but not accepted by EIP services were not considered in this paper, since they would be exempted from the new standard [12, 13].

### Participant ascertainment and inclusion criteria

Ascertainment began on 1 August 2009 for 3.5 years. We included all participants accepted by EIP services following initial assessment for suspected psychosis with the intention to offer the full 3-year intervention package of care, who met the following inclusion criteria:

1. 16–35 years old (17–35 in “Cambridgeshire North” and “Cambridgeshire South” services)
2. Resident in the catchment area, including those of no fixed abode
3. No previous contact with health services for psychotic disorder, or previous treatment with anti-psychotic medication for greater than 6 months

Four EIP services (West Norfolk, Central Norfolk, Great Yarmouth and Waveney and Suffolk) operated “extended assessment” protocols, whereby some referrals were initially offered a shorter EIP care package (up to 6 months), after which time a decision about whether to offer the full (3-year) EIP care package was taken based on clinical review. Consistent with our entry criteria and current clinical guidance [12, 13], we only included participants who were offered up to 3 years of EIP care in our incepted sample. We collected baseline sociodemographic and clinical data on all incepted participants. Participants were followed from referral until receipt of 3 years of early intervention care, or discharge from the service, if earlier. Clinical information (clinician-based and OPCRIT-based diagnoses) was obtained 6 months after acceptance and at the end of their EIP care.

### Main outcome measure

The main outcome in this study was waiting time (in days) between date of first referral and date of acceptance by EIP services. All EIP services in our study received referrals from multiple sources, including primary and secondary care providers, other tertiary mental health providers, educational establishments and self-referrals. Referral and acceptance dates were recorded in the EIP service log book. Acceptance was recorded following official acceptance onto an EIP caseload and assignment of a care coordinator; for participants initially on extended assessment but who were later upgraded to the full 3-year EIP care package, acceptance date was backdated to the date of initial acceptance. Wait days were estimated as the number of days between acceptance and referral.

### Clinical predictors

Participants who received an International Classification of Diseases, Tenth Revision (ICD-10), clinical diagnosis of psychotic disorder (F10-33), subsequently ratified by a research-based OPCRIT assessment, were classified according to their final (3-year or discharge) OPCRIT diagnosis with either a non-affective psychosis (F20-29), affective psychosis (F30-33) or substance-induced psychosis (F1X.5). OPCRIT is a 90-item symptom checklist rated from case notes to produce standardised, reliable diagnoses [17, 18]. Participants not diagnosed with an OPCRIT-confirmed FEP during their EIP care were categorised as either having an organic basis to their disorder (*N* = 17) or as “no FEP” (*N* = 94).

Using OPCRIT data obtained 6 months after EIP acceptance, we also obtained clinical measures of the presence of a psychosocial stressor prior to psychosis onset (yes/no), family history of schizophrenia (yes/no) or other mental disorder (yes/no), mode of onset (acute versus insidious, where insidious onset included ratings of “gradual” (onset over 1–6 months) and “insidious” (over 6 months) onset versus acute onset (less than 1 month)), lifetime polysubstance abuse and duration of untreated illness [DUI]. Lifetime polysubstance abuse was rated from 6 OPCRIT items relating to lifetime abuse/dependency of cannabis, alcohol or any other substance. Ratings were scored as “no lifetime abuse/dependency”, “1 substance”, “2 + substances”. DUI was initially rated in weeks, from the onset of 2 or more prodromal symptoms/signs of psychosis (including social withdrawal and impairment, peculiar behaviour, changes in affect, speech, ideation or perceptual experiences) until receipt of first treatment, including psychological therapies. We categorised DUI as follows: 0–4, 5–8, 9–12 weeks, 3–6, 7–12 and over 12 months. Using OPCRIT data collected at discharge from EIP services (3 years of care, or if discharged earlier, at the point of discharge), we rated the course of disorder as: good recovery, partial recovery or chronic course. To inspect possible changes in waiting times during the 3.5-year follow-up period, we also included a variable on calendar time, based on first referral date, and divided into six 6-month periods, from 1 August 2009. The final seventh time period was 8 months (1 Aug 2012–25 March 2013) to account for the full case ascertainment period of the study.

### Demographic predictors

We classified age-at-referral into four categories (16–19, 20–24, 25–29 and 30–35). Marital status at referral was classified as single, married/civil partnership or divorced/separated. Ethnicity was coded into 11 categories: white British, non-British white, Indian, Pakistani, Bangladeshi, Arabic, black Caribbean, black African, mixed white and black Caribbean, other mixed ethnicities and other ethnicities. We obtained parental and participant occupational data to classify our sample into standard Office for National Statistics [ONS] socioeconomic status [SES] categories [19]: professional and managerial occupations; intermediate occupations; routine and manual occupations; those not in employment (long-run unemployed, never worked, students, otherwise unclassifiable). Where data on both parents were available, we took the higher of the two. We inspected change between parental and participant SES to derive an indicator of social drift, where participants could have: a lower SES than their parents (drift), the same SES (stable), or higher SES (upward mobility). We defined country of birth as UK-born or foreign-born. Age-at-migration and years-in-the-UK were treated as categorical variables, based on a priori categories (Table 1). Finally, we distinguished between participants who had permanent accommodation at initial referral versus those of no fixed abode [NFA].

**Table 1:** Median waiting time in days between initial referral and acceptance to EIP care amongst incepted sample, by demographic characteristics

Variable	*N* (%)	Median wait time (days; IQR)	Test for difference *p* value	AIC
Age group				
16–19	243 (30.5)	18 (8, 36)	0.03^K^	2638.8
20–24	287 (36.0)	14 (6, 28)		
25–29	164 (20.5)	15 (7.5, 31)		
30–35	104 (13.0)	14 (6.5, 22.5)		
Sex				
Women	277 (34.7)	15 (8, 29)	0.78^M^	2642.8
Men	521 (65.3)	15 (7, 30)		
Ethnicity				
White, British	613 (76.8)	15 (7, 29)	<0.01^K^	2627.8
White, other	73 (9.2)	13 (6, 30)		
Mixed, white and black Caribbean	10 (1.3)	26 (12, 77)		
Mixed, white and other ethnicities	20 (2.5)	12.5 (5.5, 27)		
Indian	3 (0.4)	59 (17, 101)		
Pakistani	18 (2.3)	12.5 (7, 22)		
Bangladeshi	6 (0.8)	38 (19, 42)		
Black African	24 (3.0)	12.5 (5, 21)		
Black Caribbean	10 (1.3)	18 (3.5, 53)		
Arabic	4 (0.5)	1.5 (0.5, 4)		
Other ethnicities	17 (2l.1)	4 (0, 16)		
Country of birth				
UK-born (White British)	610 (76.4)	15 (7, 28)	0.05^K^	2639.4
UK-born (BME)	74 (9.3)	16 (7, 36)		
Foreign-born (BME)	111 (13.9)	12 (5, 29)		
Foreign-born (White British)	3 (0.4)	29 (27, 84)		
Age to the UK (Foreign-born)				
0–4 years	5 (4.4)	29 (19, 84)	0.08^K,†^	2641.3
5–9 years	17 (14.9)	13 (5, 28)		
13–19 years	34 (29.8)	10.5 (3, 34)		
20+ years	54 (47.4)	10.5 (6, 22)		
Missing data	4 (3.5)	23 (8, 37.5)		
Years in the UK (Foreign-born)				
<12 months	16 (2.0)	8 (4, 19.5)	0.29^K,†^	2643.7
1–5 years	41 (5.1)	13 (6, 36)		
>5 years	54 (6.8)	13 (6, 29)		
Missing data	4 (0.5)	23 (8, 37.5)		
Living situation				
Fixed abode	767 (96.1)	15 (7, 29)	0.52^M^	2642.7
No fixed abode	31 (3.9)	19 (7, 48)		
Marital status				
Single^a^	710 (89.0)	15 (7, 30)	0.34^K^	2643.2
Married/civil partnership	72 (9.0)	13 (7, 27.5)		
Divorced/separated	16 (2.0)	12.5 (8.5, 18.5)		
Parental SES^b^				
Professional and managerial	232 (29.1)	14.5 (6, 27)	0.48^K^	2644.6
Intermediate occupation	174 (21.8)	16 (7, 31)		
Routine and manual	217 (27.2)	15 (7, 33)		
Long-term unemployed, students and unclassifiable	175 (21.9)	16 (7, 30)		
Social drift				
Downward drift	396 (49.6)	15 (7, 30)	0.97^K^	2644.9
Stable	265 (33.2)	15 (7, 31)		
Upward mobility	137 (17.2)	14 (7, 28)		

*AIC* Akaike’s Information Criterion

^†^Including UK-born as a category

^a^Includes *n* = 8 participants with missing marital status, assumed to be single

^b^ *n* ≤ 5 participants’ parents were students at first referral, so this category was merged with the long-term unemployed and unclassified category for this analysis

^M^Mann–Whitney *U* test

^K^Kruskal–Wallis test

### Neighbourhood predictors

We geocoded participants to their residential neighbourhood at initial referral to estimate neighbourhood multiple deprivations, population density and rural–urban classification. Neighbourhoods were defined by ONS electoral wards, as previously described [16]. We estimated multiple deprivation as the proportion of households in each neighbourhood who were deprived on at least two of four deprivation domains included in the 2011 census (employment, education, health, living environment), categorised on an equal-interval scale (7.7–18; 18.1–28; 28.1–38; 38.1–47.1%). We estimated population density as the total 2011 census population in each neighbourhood divided by its area, expressed as people per square mile. We categorised population density according to the proportion of neighbourhoods: below the median (48–587 people per square mile); in the 50th–75th percentile (588–4653); 76th–95th percentile (4654–11,099); 96th–100th percentile (11,100–21,970). We used the ONS Rural–Urban Classification to define participants’ neighbourhoods as either rural, town and fringe (i.e. suburban) or urban, according to a range of routinely collected national indicators [20].

### Statistical analyses

We estimated median waiting times for all predictors with corresponding interquartile ranges (IQR), using the Mann–Whitney *U* tests or Kruskal–Wallis tests, as appropriate, to inspect univariable differences in waiting times. Next, we sought to model waiting time variation using accelerated failure time [ACF] models. These models provide an alternative parametrization to survival data, whereby changes in absolute survival time (here, in days) are estimated instead of the probability of survivorship more commonly estimated in (i.e. Cox) proportional hazards models. ACF models use a parametric approach to estimate baseline survivorship over time, which is entered as an error term in the model, and assumed to follow a known distribution; common choices for this distribution are *exponential, Weibull, normal, logistic* and *generalised gamma* distributions; their suitability can be compared via Akaike’s Information Criterion [AIC], with lower score indicating better model fit.

We set up the analysis such that entry to and exit from the follow-up period were the dates of referral and acceptance, respectively. Since ACF models are estimated using log time (i.e. log days), follow-up time (i.e. waiting time) had to be strictly positive. Where the referral and acceptance dates were identical (i.e. zero wait days; *N* = 59, 7.4%), we assigned participants an arbitrarily small positive wait day (1 day) to avoid omitting them from the analysis. We first fitted univariable ACF models for each clinical and demographic predictor, with AICs used to determine model fit in a multivariable ACF model. Next, we used a forward-fitting modelling procedure to determine the best fitting model to the data, with age, sex and ethnicity treated as a priori confounders and retained in the model irrespective of statistical significance. Other clinical and demographic predictors were retained in the model if they improved model fit, assessed via Likelihood Ratio Test [LRT]. We presented exponentiated parameter estimates on the day scale, together with 95% confidence intervals (95%CI). We fitted ACF models assuming a log-logistic distribution for baseline survivorship, which was empirically preferable to other choices (Supplemental Table 1).

Finally, to inspect whether neighbourhood predictors were associated with waiting times, we re-ran models on a subset of the cohort, excluding 31 participants of no fixed abode. We extended our ACF model to include a shared frailty term (assumed to follow a gamma distribution) at the neighbourhood level to determine whether any variation in waiting times was attributable to neighbourhood factors. We reported the size of this effect and then sought to determine whether any of the three neighbourhood factors we measured (multiple deprivation, population density or urban–rural classification) improved model fit, assessed via LRT as before. Analyses were conducted using Stata (version 13).

## Results

### Baseline characteristics

We identified 798 participants incepted into EIP services over 3.5 years, of whom 111 (13.9%) did not meet diagnostic criteria for FEP, including 17 (2.1%) participants with an organic basis to their disorder. The sample was varied in terms of baseline demographic and clinical characteristics (Tables 1, 2), with a higher proportion of men (65.3%), people under 25 years (66.5%), single persons (89.0%) and those who had experienced downward social drift compared with their parents’ SES (49.6%). Participants from a black or minority ethnic [BME] background composed 23.2% of the sample.

### Median waiting times by demographic and clinical characteristics

Fifty percent of participants were accepted within 15 days of initial referral (median = 15 days; IQR: 7 to 30). This varied according to some demographic (Table 1) and clinical (Table 2) predictors, including age (Kruskal–Wallis *p* = 0.03), ethnicity (Kruskal–Wallis *p* < 0.01), diagnosis (Kruskal–Wallis *p* < 0.01), mode of onset (Mann–Whitney *p* = 0.02) and EIP service (Kruskal–Wallis *p* < 0.01). Service-level variation in waiting times varied from a median of 8 days in the shortest service (CAMEO South; IQR 3–22) to 28 days in the longest (West Norfolk; IQR 13–89). Importantly, median waiting times did not vary significantly by several other factors (Tables 1, 2), including sex, parental SES, country of birth, duration of illness, family history of any psychiatric disorder, or course of disorder. There was initial weak evidence (*p* = 0.06) that waiting times varied by calendar period, with a trend toward longer waiting times at later time periods (Table 2).

**Table 2:** Median waiting time in days between initial referral and acceptance to EIP care amongst incepted sample, by clinical characteristics

Variable	*N* (%)	Median wait time (days; IQR)	*P* value	AIC
EIP service				
CAMEO South	191 (23.9)	8 (3, 22)	<0.01^K^	2591.0
CAMEO North	130 (16.3)	12.5 (4, 33)		
West Norfolk	51 (6.4)	28 (13, 89)		
Central Norfolk	178 (22.3)	17 (9, 28)		
Great Yarmouth and Waveney	97 (12.2)	20 (10, 31)		
Suffolk	245 (30.7)	16 (9, 29)		
FEP diagnosis				
Non-affective psychosis [F20–29]	573 (71.8)	15 (7, 28)	<0.01^K^	2636.1
Affective psychosis [30–33]	84 (10.5)	14 (6, 28.5)		
Substance-induced psychosis [F10–19]	30 (3.8)	18.5 (10, 41)		
No FEP	94 (11.8)	15.5 (10, 40)		
Organic basis to disorder	17 (2.1)	24 (16, 54)		
Mode of onset				
Acute	232 (29.1)	14 (6, 26)	0.02^M^	2637.8
Insidious	566 (70.9)	16 (7, 31)		
Premorbid functioning				
No impairment	276 (34.6)	13 (6, 28)	0.03^K^	2638.3
Impairment on 1 domain	207 (25.9)	15 (7, 29)		
Impairment on 2 domains	212 (26.6)	16 (8, 30)		
Impairment on 3 domains	103 (12.9)	19 (8, 37)		
Lifetime poly-substance abuse				
No abuse	392 (49.1)	14 (7, 28)	0.04^K^	2638.9
1 substance	171 (21.4)	15 (7, 30)		
2 or more substances	235 (29.5)	17 (9, 32)		
Psychosocial stressor prior to onset				
No	511 (64.0)	15 (7, 28)	0.11^M^	2640.4
Yes	287 (36.0)	16 (8, 31)		
Calendar time				
Aug 2009–Jan 2010	111 (13.9)	14 (5, 27)	0.06^K^	2641.3
Feb 2010–Jul 2010	140 (17.5)	16 (7.5, 27.5)		
Aug 2010–Jan 2011	121 (15.2)	14 (6, 29)		
Feb 2011–Jul 2011	93 (11.7)	14 (6, 28)		
Aug 2011–Jan 2012	95 (11.9)	14 (7, 27)		
Feb 2012–Jul 2012	112 (14.0)	17 (7, 31)		
Aug 2012–Mar 2013^a^	126 (15.8)	19 (8, 41)		
Family history of schizophrenia				
No	687 (86.1)	15 (7, 29)	0.50^M^	2642.5
Yes	111 (13.9)	15 (7, 35)		
Family history of other psychiatric disorder				
No	462 (57.9)	15 (7, 29)	0.69^M^	2642.8
Yes	336 (42.1)	16 (7, 29)		
Duration of illness				
0–4 weeks	92 (11.5)	14 (7, 25.5)	0.12^K^	2642.9
5–8 weeks	52 (6.5)	13 (6, 21)		
9–12 weeks	41 (5.1)	14 (6, 29)		
3–6 months	118 (14.8)	18.5 (10, 39)		
7–12 months	208 (26.1)	15 (6, 30.5)		
Over 12 months	287 (36.0)	15 (7, 30)		
Course of disorder				
Good recovery	350 (43.9)	15 (7, 31)	0.80^K^	2644.6
Partial recovery	236 (29.6)	15 (7, 28)		
Chronic course	212 (26.6)	16 (7, 31)		

*AIC* Akaike’s information criterion

^a^The study began on 1 Aug 2009 in CAMEO North and South, 8 September in Suffolk and 28 September in all other EIP services. The final calendar period was slightly longer than 6 months to include all referrals which presented to the four EIP services which began case ascertainment for 3.5 years in September 2009

^M^Mann–Whitney *U* test

^K^Kruskal–Wallis test

### Modelling of waiting times to EIP services

Our final multivariable model indicated that waiting times varied independently by diagnostic group, mode of onset, age, ethnicity, EIP service and calendar period, after mutual for each other and sex. With respect to diagnostic group, while no waiting time differences were observed between participants diagnosed with affective (0.5 additional wait days; 95% CI −2.0 to 2.3) or substance-induced psychoses (1.0 day; 95% CI −2.9 to 4.2) relative to non-affective psychotic disorders, participants with an organic basis to their disorder experienced significantly longer waiting times (7.3 days; 95% CI 1.0–13.2). There was also a weak trend for longer waiting times in participants without a FEP diagnosis (2.0 days; 95% CI −0.3 to 3.6; *p* = 0.09). Participants with lifetime poly-substance abuse had longer waiting times than those without such a history (1.9 days; 95% CI 0.2–3.0). Compared with people aged 16–19 at first referral, older participants appeared to have shorter waiting times (Table 3), while there was weak evidence that insidious (versus acute) onset was associated with longer waiting times (1.5 days; 95% CI −0.2 to 2.4; *p* = 0.06). After controlling for other variables, we found evidence that increasing calendar time (per 6-month period) was associated with longer waiting times (0.4 days; 95% CI 0.1, 0.6), such that the average waiting time was 4.3 days longer (95% CI 1.3, 6.2; *p* < 0.01) in the last case ascertainment period (Aug 2012–Mar 2013) than the first one (Aug 2009–Jan 2010). Calendar time fitted as a categorical variable did not improve the final model (LRT *p* = 0.62).

**Table 3:** Predictors of waiting time variation following accelerated failure time modelling, incepted sample

Variable	Unadjusted change in wait days (95% CI)	Adjusted change in wait days (95% CI)	Wald *p* value^a^
Baseline wait days^b^	15.1 (13.9, 16.4)	8.1 (5.9, 11.2)	–
FEP diagnosis			
Non-affective psychosis [F20–29]	Ref	Ref	
Affective psychosis [30–33]	−0.6 (−4.3, 3.4)	0.5 (−2.1, 2.3)	0.66
Substance-induced psychosis [F10–19]	6.1 (−1.1, 15.5)	1.0 (−2.9, 4.2)	0.58
No FEP	4.2 (−0.01, 8.8)	2.0 (−0.3, 3.6)	0.09
Organic basis to disorder	15.5 (3.4, 33.3)	7.3 (1.3, 13.2)	0.02*
Mode of onset			
Acute	Ref	Ref	
Insidious	3.1 (0.4, 5.4)	1.5 (−0.2, 2.4)	0.07
Poly-substance abuse			
No abuse	Ref	Ref	
1 substance	0.7 (−2.4, 3.7)	0.2 (−1.9, 1.6)	0.81
2 or more substances	3.7 (0.7, 6.5)	1.9 (0.2, 3.0)	0.03*
Age group			
16–19	Ref	Ref	
20–24	−3.5 (−7.1, −0.3)	−1.9 (−4.1, −0.4)	<0.01*
25–29	−1.9 (−6.0, 1.9)	−0.9 (−3.2, 0.7)	0.32
30–35	−5.3 (−9.6, −1.2)	−2.6 (−5.4, −0.7)	<0.01*
Sex			
Women	Ref	Ref	
Men	−0.5 (−3.3, 2.0)	−0.9 (−2.8, 0.3)	0.18
Ethnicity			
White, British	Ref	Ref	
White, other	−1.7 (−5.7, 2.7)	0.6 (−2.1, 2.6)	0.61
Mixed, white and black Caribbean	12.6 (−2.5, 40.4)	10.5 (1.3, 22.1)	0.02*
Mixed, white and other ethnicities	−3.4 (−9.2, 4.7)	−1.8 (−5.9, 1.6)	0.32
Indian	33.1 (−1.7, 139.6)	20.0 (−1.0, 71.5)	0.07
Pakistani	−3.0 (−9.1, 5.7)	1.1 (−3.9, 5.9)	0.65
Bangladeshi	14.8 (−2.4, 48.6)	10.6 (0.4, 24.4)	0.04*
Black African	−3.9 (−9.2, 3.0)	−0.7 (−4.7, 2.7)	0.70
Black Caribbean	−1.1 (−10.3, 16.7)	1.4 (−5.6, 10.3)	0.72
Arabic	−13.7 (−16.5, −9.0)	−6.5 (−10.4, −2.6)	<0.01*
Other ethnicities	−11.0 (−14.7, −5.5)	−5.1 (−9.0, −1.6)	<0.01*
EIP service			
CAMEO South	Ref	Ref	
CAMEO North	2.9 (−0.2, 5.7)	2.1 (−0.6, 4.1)	0.12
West Norfolk	27.0 (18.2, 37.2)	23.1 (17.6, 28.0)	<0.01*
Central Norfolk	7.7 (4.7, 10.4)	5.2 (2.9, 6.6)	<0.01*
Great Yarmouth and Waveney	9.8 (6.0, 13.4)	7.0 (4.4, 8.9)	<0.01*
Suffolk	7.5 (4.8, 9.8)	5.0 (3.1, 6.2)	<0.01*
Referral period			
Per 6-month increment (2009–2013)	0.6 (0.2, 1.0)	0.4 (0.1, 0.6)	0.01*

*p < 0.05

^a^For multivariable adjusted model

^b^In univariable analyses, the baseline group is the median wait days for the total sample in a null accelerated failure time model (univariable results) with 95% CIs. In multivariable analyses, the reported baseline median wait corresponds to the reference group in the final accelerated failure time model (i.e. White British women with acute onset non-affective psychotic disorder in CAMEO South, aged 16–19, referred in the first 6 months of the study)

**Fig. 1:**
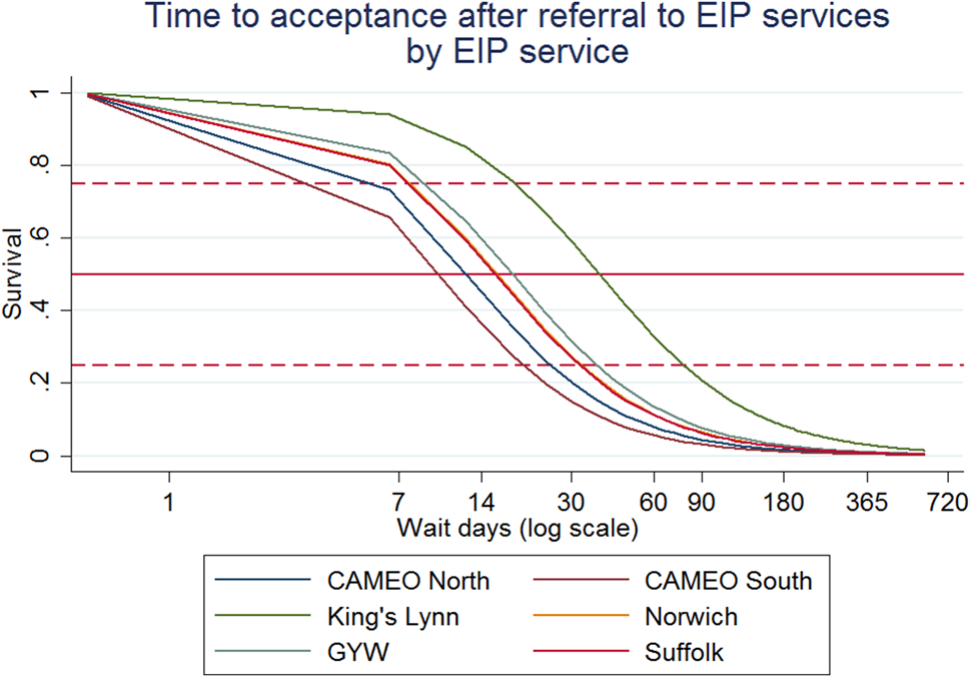
Survival curve showing variation in waiting time by EIP service. Waiting time (in days) is plotted on a logarithmic scale for clarity. Compared with waiting times in CAMEO South (Cambridgeshire) (median: 8 days; interquartile range: 3–22) waiting times were significantly longer in all other EIP services (*p* < 0.01), except CAMEO North (Peterborough), after adjustment for other covariates shown in Table 3. Median survival (waiting time) is denoted by the *horizontal solid red line, lower* and *upper* quartiles are denoted by the *horizontal dashed red lines*

We observed no differences in waiting times between white British participants and non-British white, black African, black Caribbean, Pakistani and “mixed other” ethnic minority groups, who together represented 95.0% of the sample. For the remaining participants from other BME groups, those of Arabic (−6.5 days; 95% CI −10.4, −2.6) and “other” (−5.1 days; 95% CI −9.0, −1.6) ethnicities had shorter waiting times than white British participants, while people of mixed white and black Caribbean (10.5 days; 95% CI 1.3, 22.1) and Bangladeshi (10.6 days; 95% CI 0.4, 24.4) origin showed trends toward longer waiting times. All EIP services had longer waiting times than our reference service, CAMEO South (see Table 3; Fig. 1).

Addition of a shared frailty term at the neighbourhood level did not improve model fit in a subset of the sample who could be coded to a permanent residential address at first referral (N = 767; *p* = 1.00; Supplemental Table 2). Correspondingly, we did not observe any significant variation in median waiting times by population density (likelihood ratio test [LRT] *p* = 0.26), multiple deprivation (LRT *p* = 0.53) or rural–urban classification (LRT *p* = 0.55).

## Discussion

### Principal findings

In the first investigation of waiting times in EIP services since the introduction of national guidelines to commence treatment within 2 weeks of referral for at least 50% of clients [12], our data indicated that services in a large region in the East of England were already operating close to this target (50% of clients accepted onto EIP caseloads within 15 days). Importantly, waiting times for treatment did not vary by several clinical, demographic or neighbourhood-level predictors, including sex, parental SES, marital status, country of birth, age-at-migration, years in the UK, type of FEP diagnosis, duration of illness, family history of psychiatric disorder or course of disorder. While this will be reassuring to policymakers, commissioners and EIP service providers, we noted some variation in waiting times, with a strong trend for longer waiting times over the 3.5-year case ascertainment period. In addition, longer waiting times were most consistently associated with clinical uncertainty at initial referral, including an organic basis to disorder, lifetime polysubstance abuse, insidious mode of onset or the absence of a FEP diagnosis. Of particular note, waiting times increased over the study period, and were almost a (working) week longer—on average—at the end of the study than at the start.

### Meaning of the findings

Our data suggest that EIP services do not systematically delay acceptance into care based on a variety of putative predictors. This is important since it suggests broadly equitable waiting times to EIP services following referral, irrespective of several major sociodemographic factors. Nonetheless, clinical uncertainty or more complex presentations at first referral may have resulted in longer waiting times, and such uncertainty has been associated with delays at other points on the care pathway [21]. The slightly longer waiting times observed for people aged 16–19 years also potentially fits with a pattern of clinical uncertainty, given that psychopathologies may be more diffuse at younger ages. People of mixed white and black Caribbean and Bangladeshi origin also experienced longer waiting times. A variety of reasons may underpin such variation, including potential syndromal differences at first presentation. For example, we have previously shown that people of mixed white and black Caribbean origin were at substantially increased risk of affective psychoses compared with the white British group [22].

We observed some variation in waiting times by EIP service, not explained by the neighbourhood-level characteristics of the social environment we studied. These findings accord with observations that DUP does not appear to vary at the neighbourhood level [23].

It is possible that steadily increasing waiting times observed over the study period could be the result of either demand-side factors, such as clinical or demographic changes to the casemix, or supply-side factors, such as changes to service provision, staffing levels, staffmix or resourcing and policy changes. Demand for services in our study was similar over all periods (*p* = X), although other studies have reported recent increases in incidence over time in Europe [24]. We believe it is unlikely that clinical and demographic changes to the casemix profile of participants accounted for longer waiting times over this period, given that we adjusted for all relevant predictors and a priori confounders including age, sex and ethnicity in our multivariable model. Although they could not be directly tested in this study, well-documented supply-side issues within the National Health Service have affected EIP services in England since 2009 [25, 26], including substantial financial cuts and reorganisation of EIP services. Our region was not exempt from the impact of these changes, where at least one service (Suffolk EIP) was subsumed into a more diffuse youth services model, and others underwent various aspects of reorganisation against tightening budgets. Such changes create staff shortages, morale issues and reduced service quality [25], and at least notionally, paralleled the increase in waiting times observed in our study. While further empirical research is required, it seems plausible to theorise that inadequate service resourcing would impact on several aspects of effective service delivery, including pressures to meet waiting time targets.

### Methodological considerations

Our study had a number of strengths. It was based on a large, epidemiologically complete sample. We included all clients who met precise epidemiological criteria and who were incepted into services, allowing us to present data on the full gamut of people commencing treatment under EIP care irrespective of later diagnosis. However, our findings may not generalise to other potential EIP clients, including people presenting outside our study age range (16–35 years), or people who had previously presented to mental health services for psychosis. EIP services accepted a small proportion of people who did not receive an OPCRIT-confirmed ICD-10 diagnosis of non-organic psychotic disorder (*N* = 111). Some of these participants were found to have an organic basis to their disorder at baseline (*N* = 17; 15.3%), while others received clinical diagnoses for other psychiatric conditions, including anxiety disorders (*N* = 20; 18.0%), depressive disorders (without psychosis) (*N* = 19; 17.1%) or personality disorders (*N* = 15; 13.5%). A proportion of these participants may have been at ultrahigh risk for psychosis, but EIP services in our catchment area did not routinely offer early detection at the time of the study. In England, EIP services accept clients on the basis of the initial presence of psychotic symptoms, and avoid diagnosis at first referral to allow symptoms to develop and avoid stigma in young people; this may partially contribute to the proportion of non-psychotic psychiatric morbidity in this sample. Our EIP services served populations which were broadly representative of the English population in terms of age, sex and multiple deprivation [16], although they were more rural and less ethnically diverse than elsewhere in England. Although we found no evidence that waiting times varied by population density or deprivation, there was some variation by ethnicity, which may have implications for EIP services operating in populations with a higher proportion of ethnic minority groups. We used appropriate statistical models to investigate several clinical, demographic and neighbourhood-level predictors. All OPCRIT raters received training prior to assessment, with good inter-rater reliability for resultant ICD-10 diagnose [16]. Nonetheless, we recognise that some clinical predictors rated from OPCRIT relied on single-item variables, including duration of illness, which may have introduced some measurement error into our models. We did not collect data on duration of untreated psychosis.

Referral and acceptance dates were recorded in each EIP service’s log book, which are strictly maintained for NHS auditing and routine statistical reporting, meaning the data should be reliable. We were unable to collect additional data about the referral pathway prior to presentation to EIP services, meaning total waiting times within the entire healthcare system may have been longer than those reported here. An implicit assumption of our study was that the recorded acceptance date was concomitant with assignment to a care coordinator and receipt of a NICE-concordant package of EIP care, although we could not validate this. Other studies have shown that further treatment delays may arise at other points in the care pathway [27, 28]; opportunities to investigate the core components of EIP waiting times and treatment delays should become available in England following the introduction of the new Access and Waiting Time Standard [12, 13] and routine benchmarking of these data.

We did not have ethical approval to record data on participants who were referred to, but not accepted by EIP services (*N* = 207), and so we were unable to determine the length of time it took services to reach an acceptance decision on these participants. While such patients are exempt from the current access and waiting time standards, they would have undergone a period of assessment with EIP services which both delays any subsequent referral and treatment within the mental healthcare system and consumes finite EIP resources. One important change introduced by the new access and waiting time standard in England is the change in the upper age limit for referrals to EIP services from 35 to 64 years [12, 13]. While this will reduce implicit age–sex biases inherent with a cutoff of 35 years, given the underlying epidemiology of FEP [29], prediction models suggest that this will increase EIP caseloads by up to 46% [30]. Our study results do not generalise to these older groups per se, but the additional demand on EIP services may undermine their ability to meet current waiting time targets across all ages without appropriate additional resourcing. Further research is urgently required to establish whether people who present with FEP after 35 years have more or less severe psychopathologies than those hitherto accepted by EIP services. Nonetheless, our data highlight those clinical characteristics at first presentation which may delay acceptance into a NICE-concordant package of care in young adults, and this should serve to further reduce waiting times in EIP care.

## Electronic supplementary material

Below is the link to the electronic supplementary material.

Supplementary material 1 (DOCX 16 KB)
