# Supplementary material for: Waiting time variation in Early Intervention Psychosis services: longitudinal evidence from the SEPEA naturalistic cohort study
Source: Soc Psychiatry Psychiatr Epidemiol. 2017 Feb 18;52(5):563–74. doi: 10.1007/s00127-017-1343-7 (PMC5423995; doi:10.1007/s00127-017-1343-7)
Supplement: Supplementary file 1 — Supplementary material 1 (DOCX 16 KB) [file 127_2017_1343_MOESM1_ESM.docx]

**Supplementary Tables for:**

**Waiting time variation in Early Intervention Psychosis Services: longitudinal evidence from the SEPEA naturalistic cohort study**

Kirkbride JB Ph.D.,^1 2^ Hameed Y MRCPsych,^3^ Wright L B.Sc. (Hons),^3^ Russell K M.Sc.,^3^ Knight C,^4^ Perez J Ph.D.,^2 4^ Jones PB FMedSci ^2 4^

^1^PsyLife group, Division of Psychiatry, UCL, London, W1T 7NF, UK

^2^Department of Psychiatry, University of Cambridge, Cambridge, CB2 0SZ, UK

^3^Norfolk & Suffolk Foundation Trust, Norwich, Norfolk, NR6 5BE, UK

^4^Cambridgeshire & Peterborough Foundation Trust, and NIHR Collaboration for Leadership in Applied Health Research and Care (CLAHRC) East of England, Cambridge, Cambridgeshire, CB21 5EF, UK

*Corresponding author: Dr James Kirkbride [j.kirkbride@ucl.ac.uk](mailto:j.kirkbride@ucl.ac.uk)

**Supplemental Table 1: Accelerated failure time model fit indices for different distributional assumptions about the baseline survival function**

| Model^1^ | AIC^2^ |
| --- | --- |
| Gamma |  |
| Exponential | 2588.0 |
| Weibull | 2588.0 |
| Log-normal | 2617.3 |
| Log-logistic | **2571.9** |

^1^All models are fitted with the covariates from the final model presented in Table 3

^2^Lower score indicates better model fit. Best-fitting model highlighted in bold.

**Supplemental Table 2: Multilevel modelling of variance in waiting times in an accelerated failure time model fitted with shared frailty at the neighbourhood level**

| Variable | N (%) | Median wait days (IQR) | LRT p-value^1^ |
| --- | --- | --- | --- |
| Theta (null model)^2^ | 767 (100) | 0.00 | 1.00 |
| Theta (final model)^2^ | 767 (100) | 0.00 | 1.00 |
| Neighbourhood population density (People per square mile) |  |  | 0.26 |
| 48-587 (Below median) | 162 (21.1) | 14.5 (7, 27) |  |
| 588-4,653 (50-75^th^ percentile) | 206 (26.9) | 15 (7, 34) |  |
| 4,654-11,099 (76-95^th^ percentile) | 258 (33.6) | 16 (7, 32) |  |
| 11,100-21,970 (96-100^th^ percentile) | 141 (18.4) | 14 (7, 25) |  |
| Neighbourhood multiple deprivation (% households) |  |  | 0.53 |
| 7.8-18.0% | 188 (21.1) | 14 (5, 28) |  |
| 18.1-28.0% | 338 (44.1) | 14.5 (7, 27) |  |
| 28.1-38.0% | 186 24.3) | 16 (7, 37) |  |
| 38.1-47.1% | 55 (7.2) | 18 (10, 35) |  |
| Rural-Urban classification |  |  |  |
| Rural | 96 (12.5) | 15 (7, 28) | 0.55 |
| Suburban | 109 (14.2) | 14 (7, 28) |  |
| Urban | 562 (73.3) | 15 (7, 30) |  |

^1^Likelihood ratio test p-values for each neighbourhood variable entered into full, final model presented in Table 3. Model restricted to N=767 since N=31 were of no fixed abode and could not be assigned to a neighbourhood.

^2^In a shared frailty model, theta reports the level of shared frailty attributable to neighbourhood clustering. In both the null and fully adjusted models, no shared frailty was observed, indicating an absence of neighbourhood effects on waiting times.
